# Geographic inequalities in health intervention coverage – mapping the composite coverage index in Peru using geospatial modelling

**DOI:** 10.1186/s12889-022-14371-7

**Published:** 2022-11-17

**Authors:** Leonardo Z. Ferreira, C. Edson Utazi, Luis Huicho, Kristine Nilsen, Fernando P. Hartwig, Andrew J. Tatem, Aluisio J. D. Barros

**Affiliations:** 1 International Center for Equity in Health, Universidade Federal de Pelotas, R. Marechal Deodoro, Centro, Pelotas, 1160 Brazil; 2 Post-Graduate Program in Epidemiology, Universidade Federal de Pelotas, Pelotas, Brazil; 3 WorldPop, Geography and Environmental Science, University of Southampton, Southampton, UK; 4 Centro de Investigación en Salud Materna E Infantil, Universidad Peruana Cayetano Heredia, Lima, Peru; 5 Centro de Investigación para El Desarrollo Integral Y Sostenible, Universidad Peruana Cayetano Heredia, Lima, Peru; 6 School of Medicine, Universidad Peruana Cayetano Heredia, Lima, Peru

**Keywords:** Geospatial modelling, Child health, Woman’s health, Composite coverage index, Peru

## Abstract

**Background:**

The composite coverage index (CCI) provides an integrated perspective towards universal health coverage in the context of reproductive, maternal, newborn and child health. Given the sample design of most household surveys does not provide coverage estimates below the first administrative level, approaches for achieving more granular estimates are needed. We used a model-based geostatistical approach to estimate the CCI at multiple resolutions in Peru.

**Methods:**

We generated estimates for the eight indicators on which the CCI is based for the departments, provinces, and areas of 5 × 5 km of Peru using data from two national household surveys carried out in 2018 and 2019 plus geospatial covariates. Bayesian geostatistical models were fit using the INLA-SPDE approach. We assessed model fit using cross-validation at the survey cluster level and by comparing modelled and direct survey estimates at the department-level.

**Results:**

CCI coverage in the provinces along the coast was consistently higher than in the remainder of the country. Jungle areas in the north and east presented the lowest coverage levels and the largest gaps between and within provinces. The greatest inequalities were found, unsurprisingly, in the largest provinces where populations are scattered in jungle territory and are difficult to reach.

**Conclusions:**

Our study highlighted provinces with high levels of inequality in CCI coverage indicating areas, mostly low-populated jungle areas, where more attention is needed. We also uncovered other areas, such as the border with Bolivia, where coverage is lower than the coastal provinces and should receive increased efforts. More generally, our results make the case for high-resolution estimates to unveil geographic inequities otherwise hidden by the usual levels of survey representativeness.

**Supplementary Information:**

The online version contains supplementary material available at 10.1186/s12889-022-14371-7.

## Introduction

Peru has shown tremendous progress in improving the health and survival of women and children in the past few decades [1]. Under-five mortality rates and under-five stunting prevalence dropped by over 50% since 2000, mainly due to the increase in the coverage of reproductive, maternal, newborn and child health (RMNCH) indicators, better water and sanitation conditions, along with improvements in the social determinants of health, especially among the poorest [2]. These efforts elevated the country to a prime position in the pursuit of universal health coverage (UHC) in terms of access to the full range of quality health services without undue financial burden, although substantial inequities remain, which need to be explored in further detail up to the local levels.

The Peruvian health system is fragmented into a) the Health Insurance System (SIS), a public scheme that provides subsidized curative and preventative health services to the unemployed and to informally employed segments of the population not entitled to other schemes, b) the Social Security (ESSALUD), a public scheme financed by employers and employees that covers the sector of formal employees with curative and preventive services, and c) the private sector. Although the access and coverage gap between urban and rural areas and by wealth have decreased significantly overtime in Peru, the indigenous communities still represent one of the most neglected groups, even if there are efforts to address their needs with special interventions [3].

Since moving towards UHC on RMNCH relies on a broad set of services and interventions, monitoring its progress requires data on multiple indicators. And even overlooking the difficulty of reporting substantial amounts of data, visualizing and advocating for dozens of indicators hampers the prioritization of areas and subgroups that are farther from receiving a comprehensive assistance. In order to account for several indicators and present an integrated summary measure, the composite coverage index (CCI) was created and has been widely used as a proxy for tracking UHC in low- and middle-income countries in the context of RMNCH [4–6]. The CCI is a weighted average of eight essential preventive and curative interventions along the continuum of care, covering four stages including reproductive, pregnancy, newborn and child health. Its composition has proven to be robust as the inclusion of other important interventions have shown to have little impact in the estimates [7]. Also, its strong associations with under-five mortality rates and stunting further support that the CCI can capture adequately the combined effect of health interventions [8].

Like the majority of low- and middle-income countries, Peru relies heavily on information from national health surveys to monitor the progress of many RMNCH interventions, allowing data-driven actions to increase coverage and reduce inequalities [9–11]. These actions are more effective when stakeholders and policy planners have available data disaggregated at local levels, where policy is ultimately implemented [12, 13]. However, the sampling design of the national surveys only provides reliable estimates for large subnational divisions, as further geographical disaggregation would require much larger (often prohibitively so) sample sizes. Alternatively, indirect estimates can be derived for smaller areas using geospatial modelling approaches, as previous studies have done for RMNCH outcomes in recent years [14–16]. These strategies combine the georeferenced data from the surveys with relevant geospatial covariates, while also taking advantage of spatial correlation, to predict small area estimates.

While a few studies have generated estimates for individual interventions or health outcomes at global scale in which Peru was included, such as malnutrition [17], mortality [18] and diarrhoea management [19], no studies on RMNCH interventions were exclusively focused on Peru. Based on fine-scale estimates generated using geospatial modelling techniques, this study aims to describe the CCI coverage at province and grid-level in Peru, enabling local managers to identify and act on areas in need of prioritization.

## Methods

The following sections describe each stage of the modelling process. Further details can be found in the supplementary materials.

### Study area

Peru is an upper-middle income country located in the South American continent. Its lands cover around 1.28 million km^2^ making it the 19^th^ largest country in the world with a total population of nearly 31 million [20]. It shares borders with Ecuador, Colombia, Brazil, Bolivia, Chile, and the Pacific Ocean. The country is divided into 25 first administrative units (24 departments plus the Callao province) which are subdivided into 196 provinces, and further into districts. The geography of Peru is often divided into three main ecological zones known as a) coast, a semiarid margin bordered by the Pacific Ocean, b) highlands or the Andean mountains, a climatic diverse area separating the other two ecological zones from north to south, and c) jungle which is the most extensive zone mostly covered by the Amazon rainforest [21].

### Composite coverage index data

Carried out annually since 2004, the Encuesta Demográfica y de Salud Familiar (ENDES) is a household survey designed to provide estimates at the national and departmental levels for several health and nutritional indicators for women and children in Peru. The 2018 ENDES survey carried out a multi-stage sampling process by selecting 3,254 enumeration areas (EAs), or primary sampling units (also known as clusters – the unit of analysis in this study), proportionally distributed in all departments, followed by 36,760 households in the second stage. Similarly, the 2019 survey sampled 36,745 households in 3,254 EAs. More details on the sampling methodology can be found in the surveys’ reports [22, 23].

**Fig. 1:**
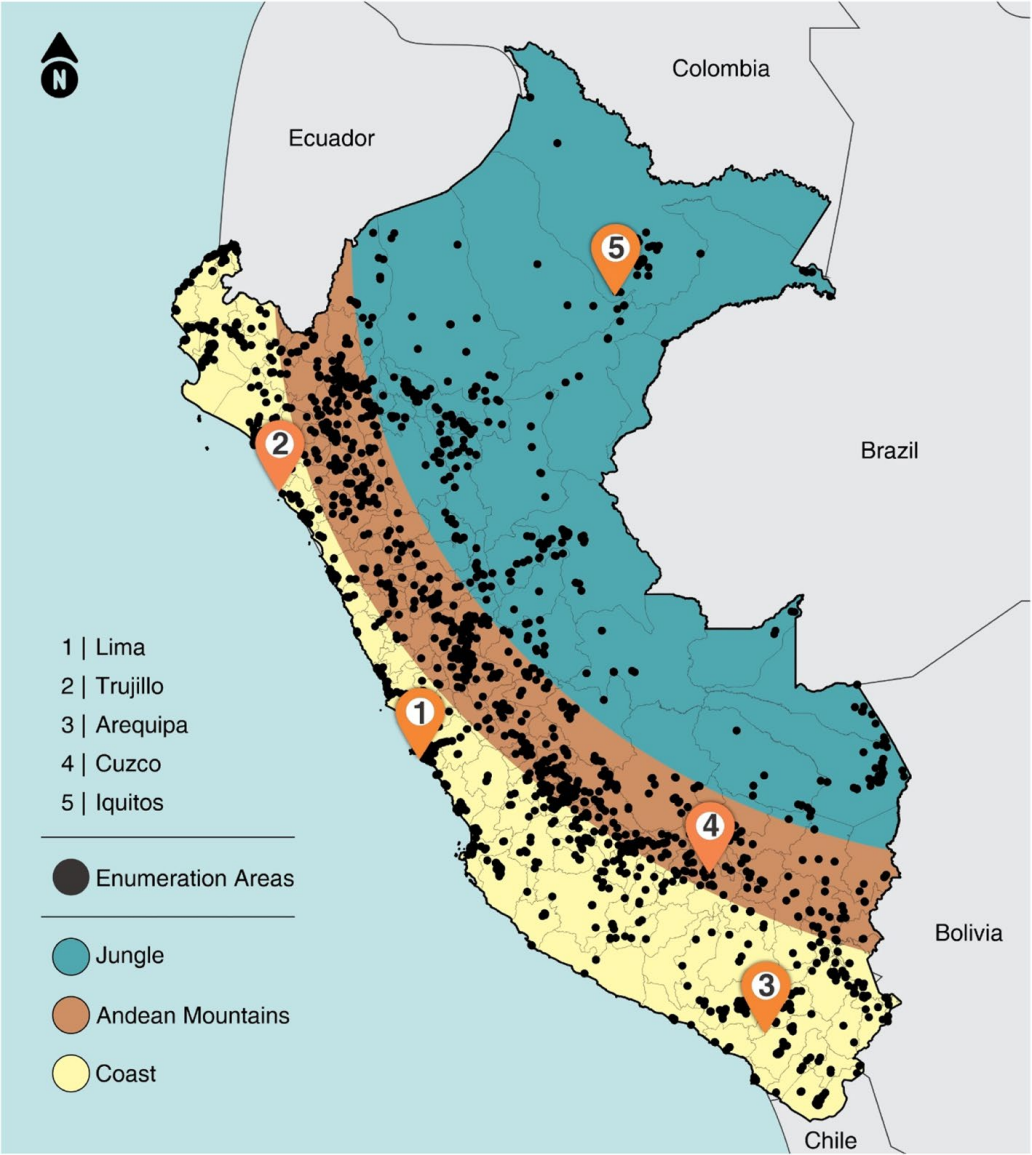
Map of the ecological zones of Peru and the distribution of cluster locations for ENDES 2018 and 2019 surveys

To increase the sample sizes, we combined data from the 2018 and 2019 ENDES surveys (Fig. 1) and used them to calculate each of the indicators that are part of the CCI. The CCI is a weighted average of coverage with eight essential maternal and child health interventions and comprises the four stages of the continuum of care. Its formula is given by

1 $$CCI=\frac14\left(DFPSm+\frac{ANC4+SBA}2+\frac{BCG+2\left(DPT3\right)+MSL}4+\frac{ORS+CAREP}2\right)$$

where the interventions are: demand for family planning satisfied with modern methods (DFPSm), at least four antenatal care visits (ANC4), skilled birth attendant (SBA), one dose of bacille Calmette-Guérin vaccine (BCG), three doses of diphtheria-pertussis-tetanus vaccine (DPT3), one dose of measles vaccine (MSL), oral rehydration salts for diarrhoea (ORS), and care-seeking for suspected childhood pneumonia (CAREP) [7]. The complete definition on the calculation of each indicator can be found in the supplementary table 1.

The coordinates for each cluster are displaced by up to 2 km in urban areas and 5 km in rural areas to protect the anonymity of the respondents. This displacement was considered in the covariate extraction process by drawing 2 km buffers for urban areas and 5 km buffers for rural areas, taking their mean accordingly [24].

### Geospatial covariates and covariate selection

A suite of 14 covariate layers known to correlate directly or indirectly with RMNCH coverage indicators were considered as predictors for each of the eight modelled indicators. These covariates include measures of accessibility, remoteness, urbanicity, and sociodemographic characteristics that were found to be associated with health interventions in previous studies [15, 25–27]. Some of these covariates were surfaces obtained from satellite imagery and publicly available repositories while others were interpolated using survey and health facility data points. Further information for each of the covariates is found in the supplementary tables 2 and 3.

We carried out a covariate selection strategy divided in two stages to achieve the best fit without overparameterizing the models: 1) testing the predictors seeking the best outcome-covariate relationship, followed by 2) backward elimination process within a stepwise logistic regression, where variables were dropped starting with the ones with the highest p values until none had a p value greater than 5%. Fractional polynomials of up to the second order were tested for all predictors, as well as the logarithmic transformation, to allow for model flexibility and capturing of non-linear relationships. We also assessed the association between all covariates using Pearson’s correlation before the stepwise selection, where we chose a single covariate with the highest association to the outcome to address the problem of (multi)collinearity, for pairs with a high correlation coefficient (> 0.8).

### Geospatial model

We followed a model-based geostatistical approach [28], similar to what was done previously [29, 30], to predict each indicator and the CCI at 5 × 5 km resolution in Peru, leveraging geospatial covariate information and spatial correlation in the data. We fitted eight different models, one for each indicator, and combined their posterior distributions to obtain estimates for the CCI. Let $Y\left({s}_{i}\right)$ be the number of individuals with a given outcome at cluster location ${s}_{i}$ (i = 1, …, n), out of a total of $N\left({s}_{i}\right)$ individuals sampled at the location. The model can be defined as:

2 $$\begin{array}{c}Y\left({s}_{i}\right) \sim Binomial (N\left({s}_{i}\right), p\left({s}_{i}\right))\\logit\left(p\left({s}_{i}\right)\right)=x{\left({s}_{i}\right)}^{T} \beta + \omega \left({s}_{i}\right)+\epsilon ({s}_{i})\end{array}$$

where $x({s}_{i})$ is a set of covariates values associated with cluster ${s}_{i}$, $\beta$ are the corresponding regression parameters, $\omega \left({s}_{i}\right)$ is a Gaussian spatial random effect used to capture residual spatial correlation in the model, and $\epsilon ({s}_{i})$ is a Gaussian random effect used to model non-spatial residual variation. The geostatistical model described above was fitted in a Bayesian framework using the integrated nested Laplace approximation with the stochastic partial differential equations [31].

We drew 1000 samples from the posterior predictive distribution generated from each model and combined them using the CCI formula. Then, we summarized the resulting posterior distribution to produce estimates of the CCI over a grid of 5 × 5 km covering the entire study area. The estimates were further aggregated to the first and the second administrative divisions through weighting using gridded population data.

Throughout, we present uncertainty estimates from the posterior distributions as the width of the 95% credible intervals (i.e. the difference between the 97.5 and the 2.5 percentiles).

### Model validation

The validity of the estimated models was assessed using an out-of-sample cross validation strategy. Data from all indicators were divided into five folds to ensure a minimum sample size of 50 clusters within each fold. We calculated and presented the following metrics: bias (mean error), the magnitude of the error (mean absolute error—MAE) and the correlation between predicted and observed values. We also compared predicted estimates aggregated at the first administrative division to the observed estimates directly derived from the surveys.

We used Stata 16 [32] for survey data analysis and the covariate selection process and R 4.0.2 [33] for the processing of geospatial covariates, model fitting and validation.

## Results

Overall, the national coverage of the CCI in the country is 71.6% (Table 1). Interventions like SBA and BCG vaccination are nearly universal in Peru with coverage above 95% (Table 1). On the other hand, treatment of diarrhoea using ORS is surprisingly low with only 33.6% (Table 1). Sample sizes are large for pregnancy and reproductive health indicators, moderate for vaccines and low for treatment of childhood illnesses.

**Table 1:** Description of the CCI and its indicators in the sample

Indicator	Number of clusters	Number of individuals	National coverage
Skilled birth attendant	6,383	24,513	94.6%
Antenatal care 4 + visits	6,383	24,141	96.3%
Demand for family planning satisfied with modern methods	5,296	12,059	65.5%
DPT3 vaccine coverage	4,861	8,706	85.3%
BCG vaccine coverage	4,861	8,706	95.3%
Measles vaccine coverage	4,861	8,706	80.0%
Oral rehydration salts for diarrhoea	3,301	5,014	33.6%
Care-seeking for suspected pneumonia	1,639	1,956	70.1%
Composite coverage index	-	-	71.6%

Out of the 14 geospatial covariates, improved sanitation coverage was the most stable predictor as it was selected in 7 out of the 8 indicators, followed by the mean number of years of education for women, used in six indicators. Conversely, improved water coverage failed to remain as a predictor in all models and was left out of analysis. Both travel time to health facilities and urbanicity were only eligible for one model each, putting them among the least relevant covariates. The median number of covariates used to fit the models was seven, with SBA using 10 covariates and CAREP fitting the final model with a single predictor (Supplementary Table 4).

**Fig. 2:**
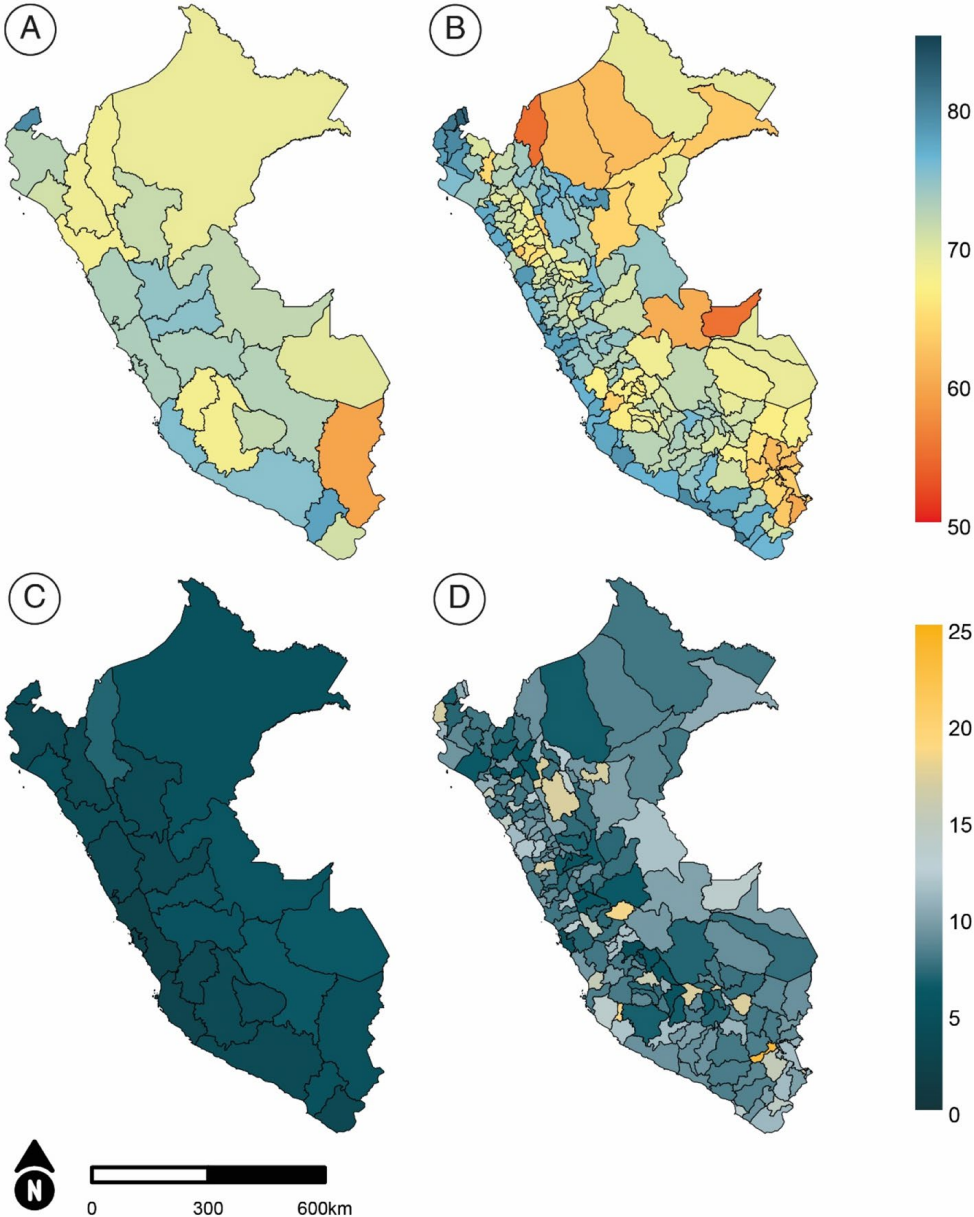
Geographical distribution of CCI coverage in Peru for **a**) the 25 departments **b**) the 196 provinces and associated uncertainty measured as **c**) the width of the 95% confidence intervals for departmental estimates and, **d**) the width of the 95% credible intervals for provincial estimates

The geospatial estimates for CCI coverage at the first and second administrative divisions of Peru (departments and provinces, respectively) are presented in Fig. 2. Coverage ranged from 59.6% in Puno, in the south-east of the country, to 79.1% in Tumbes, in the north-west (Fig. 2A). Figure 2B shows a clear pattern of higher coverage for the provinces along the coast while most of the provinces with the lowest coverages are in the jungle area. Also, substantial disparities in coverage are observed between the provinces within each department. The maximum difference between the CCI coverage in provinces of Madre de Dios, Tumbes and Ica range from 1 percentage point (p.p.) to 2.3 p.p, while these differences between the provinces of Amazonas and Ucayali go up to 20 p.p. The widest gaps between provinces are found in jungle departments but important gaps can also be seen in several departments along the coast, such as Piura, La Libertad, Ancash, and Lima.

The same way estimates from sampled data present variability, the estimates generated by the geospatial models are sensitive to the amount of data available in each area, and the further we disaggregate, the more uncertainty we may observe. Figure 2C presents the width of the confidence intervals as a measure of uncertainty for the estimates at the first administrative division that were directly derived from the surveys. The width of the credible intervals for each of the provincial estimates generated by the geospatial models are shown in Fig. 2D. The median width of the credible intervals for the provinces is 8.6 p.p., meaning that at least half of the estimates should vary no more than 4.3 p.p. around the point estimate (median coverage of 71.7%). However, estimates for the provinces coloured in orange should be interpreted with more caution since those estimates could lay within a 15 to 20 p.p. interval, with a maximum interval width of 23 p.p.

**Fig. 3:**
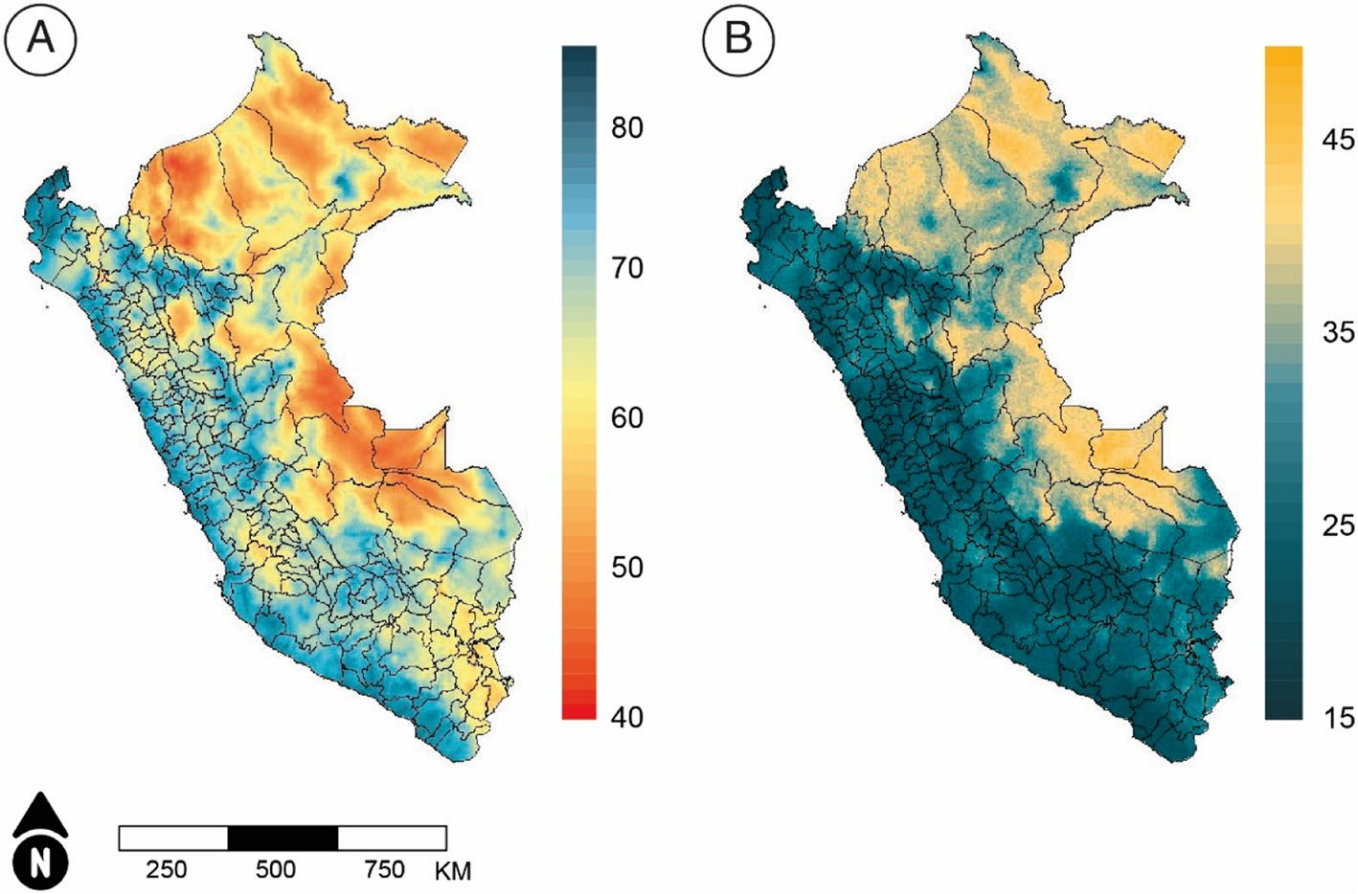
**a)** Geographical distribution of the CCI in Peru by 5 × 5 km, and **b**) associated uncertainty map measured as the width of the 95% credible intervals

Geospatial models can provide estimates for much smaller areas than the political administrative divisions of a country. Using grids of high resolution can shed light on pockets of low or high coverage otherwise masked by aggregation that may hide the most vulnerable subgroups of the population. Figure 3 presents high-resolution estimates in grids of 5 × 5 km along with uncertainty maps measured using the width of the credible intervals. Pockets of low coverage can be seen in several provinces across the jungle, while some smaller pockets exist along the highlands throughout the country. These estimates show a more detailed scenario, although increasing the resolution also increases their uncertainty, as presented in Fig. 3B. Of note, Figs. 2B and D are not directly comparable to Fig. 3 because scales differ.

The correlation of the predicted coverage against the observed cluster estimates was strong for SBA, weak for CAREP and moderate for the remainder. Bias was close to zero for all indicators and the magnitude of the errors was between 4 and 7 percentage points for BCG, SBA and ANC4, around 20 for DFPSm, MSL and DPT, and close to 30 for CAREP and ORS. We also generated estimates at the departmental-level and compared them to the published survey results, where the mean difference at the departmental-level for the CCI was 1.7% and the largest difference was 5.2% in Madre de Dios. All validation metrics and further details on the validations process are available in the supplementary tables 5 and 6.

## Discussion

Here, we produced fine-scale estimates for the CCI using geospatial models for assisting researchers and stakeholders in unveiling hidden areas and populations in need of prioritization in the context of RMNCH [14]. Through these models, it is possible to obtain information that is not available using conventional methods and can be easily employed for health policy planning and decision-making. When comparing department-level estimates to province-level estimates, it is evident that very different coverage levels exist throughout the country, and even more detailed patterns are observable when moving down to the grid-level.

In general, the size of the provinces in Peru seems sufficient to accurately inform the local context in most of the country. However, this may not be true when looking at larger provinces predominantly located in the Amazon rainforest. Some of these provinces, especially in the north and east of Peru, do not stand out as low coverage in the province-level coverage map, mainly because the low coverage areas are less populated and, on average, province coverage is not particularly low. With the high-resolution map, it is possible to identify areas with very low coverage within such provinces, supporting the use of both maps as complementary resources since the provinces account for the size of the population affected and the grids focus on anyone that lives on a specific zone. By this means, local managers will have their attention drawn to these places with enough information to verify what type of action is most needed.

The rationale behind choosing the CCI rather than one or several of the many essential RMNCH interventions is simple – one composite indicator gives a broad perspective of the status of health intervention coverage [7]. Looking at antenatal care or treatment of childhood diseases instead, we found distinct levels of coverage without marked geographical variation. These patterns are completely different from indicators such as demand for family planning satisfied or skilled birth attendant – both presented huge spatial heterogeneity with pockets of low and high coverage. As a composite measure, the CCI is able to highlight areas and subgroups that are struggling in multiple fronts and are far away from UHC. Additionally, being a weighted average, it is less susceptible to imprecision of some specific indicator.

Peru was able to improve substantially its RMNCH indicators and the availability and quality of the departmental level data through annual ENDES to monitor appropriately the progress of coverage and impact indicators throughout the latest few decades [2, 34]. Now, it faces the challenge of sustaining such a progress by tracking reliably the evolution of interventions coverage at the provincial and more local levels. The use of granular information like the CCI based on geospatial methods and high-resolution mapping may allow an increased efficiency of policy makers in the design, implementation and impact evaluation of programs and interventions for further improvement of maternal and child health, with a special focus on the most disadvantaged communities, which are located mainly within the Amazon and the Andean provinces. Of note, the pockets of lowest CCI coverage identified in our study are within provinces with the lowest human development index [35], which are also more rural, concentrate a higher proportion of indigenous communities, have lower density of human resources for health, more precarious infrastructure and lower transport and internet connectivity, and lower access to water and sanitation [36, 37]. These are also the areas that face the greatest challenges in terms of local governance and policy development capacity [38]. Thus, our results highlight the need to rethink planning and implementation of interventions in Peru, by paying particular attention to the neediest areas that are lagging behind and by taking into account the current status of the diverse determinants of health in such areas.

Some limitations should be considered when interpreting the estimates described in our study. All modelled estimates carry uncertainty, which should be observed when interpreting the coverage estimates. Due to low sample sizes, indicators for remote and less populated areas as well as those related to treatment of childhood diseases can be unstable, as they are based on information from few clusters that depend on children presenting with pneumonia or diarrhoea at the time of the survey. The most critical areas were concentrated in the Amazon jungle where population density is low, and many preservation areas exist. Also, increased granularity implies greater uncertainty. This phenomenon is evident when comparing the uncertainty produced in the different levels of aggregation.

## Conclusions

In summary, our study presents CCI coverage at three sub-national levels in Peru, pinpointing where are the population segments with the lowest coverage levels. It also showcases the importance of geospatial methods and high-resolution mapping in comparison to coverage estimates at administrative division level, especially where the divisions cover a large area and are highly heterogeneous. Our results constitute a valuable guide for local policy makers and managers to focus efforts in disadvantaged areas.

## Supplementary Information

**Additional file 1.**

## Data Availability

Data on the national household surveys can be obtained from the Instituto Nacional de Estadística e Informática (http://iinei.inei.gob.pe/microdatos). Data sources from the covariates are listed in the supplementary material. Codes and further data can be obtained directly from the authors.
