# Supplementary material for: Geographic inequalities in health intervention coverage – mapping the composite coverage index in Peru using geospatial modelling
Source: BMC Public Health. 2022 Nov 17;22:2104. doi: 10.1186/s12889-022-14371-7 (PMC9670533; doi:10.1186/s12889-022-14371-7)
Supplement: Supplementary file 1 — Additional file 1. [file 12889_2022_14371_MOESM1_ESM.docx]

# Geospatial modelling process

## Covariate preparation and selection

After obtaining all covariates listed in the Supplementary Table 2, these were standardized in the WGS84 EPSG coordinate reference system using lat/long projection and aggregated to the 5 x 5 km resolution through bilinear interpolation using the raster package in R. When the covariate data were extracted for each cluster location, we drew 2 km buffers in urban areas and 5 km buffers in rural areas to account for the displacement added to protect the anonymity of the respondents. We used only continuous variables and extracted the weighted mean of all cells within the buffer radius for the analysis. We tested the association between all covariates using Pearson’s correlation to prevent any issues with multicollinearity. Then, we performed a backwards elimination process using a binomial logistic regression allowing for fractional polynomials of the up to the second order to find the best fit for each covariate. We kept only the covariate with the highest association (highest z-value in a bivariate analysis) to the outcome when two or more highly correlated covariates (rho>0.8) were selected for a single model.

## Model fit details

We used a geostatistical model based on the equation presented in the main paper fitted in a Bayesian framework using the INLA-SPDE approach with non-informative $N({\boldsymbol{0},10}^{6}\boldsymbol{I})$ priors. The SPDE model used penalized complexity priors with alpha set to 2, the spatial range of the field set to (0.01, 0.92 (5% of the north-south extent of the country)), and the standard deviation of the field set to (3, 0.01). The mesh was constructed using the cluster coordinates, country boundaries, max edge of 0.05 for inner triangles, 0.6 for outer triangles and a cut-off of 0.1. Afterwards, it was assessed through visual inspection. For each model, we generated 1000 samples from the posterior distribution for the predicted location at 5 x 5 km grid cell resolution. These estimates were further aggregated at the first and second administrative divisions using the boundaries obtained from GADM (https://gadm.org), weighted by the population estimates obtained from Worldpop (www.worldpop.org).

Supplementary table 1 - Complete definitions for the composite coverage index (CCI) and its indicators

| **Indicator** | **Acronym** | **Numerator** | **Denominator** |
| --- | --- | --- | --- |
| Demand for family planning satisfied by modern methods | FPSm | Who is using (or whose partner is using) a modern contraceptive method | Women aged 15-49 years either married or in union in need of contraception |
| Antenatal care 4 or more visits | ANC4 | Attended at least four antenatal care (ANC) visits with any provider | Women aged 15-49 years who had a birth in the last 3 years before the survey |
| Skilled attendant at delivery | SBA | Delivered by a skilled attendant (based on each country’s definition of skilled attendant) | Women aged 15-49 years who had a birth in the last 3 years before the survey |
| BCG vaccination | BCG | Received Bacillus Calmette-Guérin (BCG) vaccine | All live-children, 12-23 months |
| DPT3 vaccination | DPT3 | Received 3 doses of Diphteria, Pertussis, Tetanus (DPT) vaccine | All live-children, 12-23 months |
| Measles vaccination | MSL | Received measles vaccine | All live-children, 12-23 months |
| Treatment for diarrhea | ORS | Received oral rehydration salts (ORS) | All live children aged 0-59 months with diarrhea in the last 2 weeks |
| Care-seeking for pneumonia | CAREP | Sought treatment from an appropriate health facility or provider. | Live children, 0-59 months, suspected pneumonia in the last 2 weeks |
| Composite coverage index | CCI | 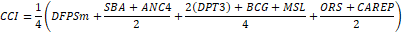 | |

Supplementary table 2 – Description of covariates included in the analysis

| **Covariate** | **Year** | **Resolution** | **Unit** | **Source** |
| --- | --- | --- | --- | --- |
| Altitude | NA | 1km | meters | SRTM (raster package) |
| Travel time to cities >50,000 | 2000 | 1km | minutes | ftp://ftp.worldpop.org.uk/GIS/Covariates/Global_2000_2020/PER/ |
| Distance to health facilities | 2017 | 1km | meters | https://www.datosabiertos.gob.pe/dataset/minsa-ipress |
| Enhanced vegetation index | 2017 | 1km | 0 to 10000 (least to most vegetation) | https://ladsweb.modaps.eosdis.nasa.gov/search/history |
| Urbanicity | 2014 | 1km | 0.00 to 1.00 (extremely rural to urban) | https://jeodpp.jrc.ec.europa.eu/ftp/jrc-opendata/GHSL/GHS_BUILT_LDSMT_GLOBE_R2018A/ |
| Nighttime lights | 2016 | 100m | nW/cm2/sr | ftp://ftp.worldpop.org.uk/GIS/Covariates/Global_2000_2020/PER/ |
| Improved water coverage | 2018 | 1km | proportion | Interpolated using kriging |
| Improved sanitation coverage | 2018 | 1km | proportion | Interpolated using kriging |
| Mean number of household members | 2018 | 1km | number | Interpolated using kriging |
| % of households in Q1 or Q2 | 2018 | 1km | proportion | Interpolated using kriging |
| Mean women's years of education | 2018 | 1km | proportion | Interpolated using kriging |
| % of indigenous population | 2018 | 1km | proportion | Interpolated using kriging |
| Distance to protected areas | 2017 | 100m | km | ftp://ftp.worldpop.org.uk/GIS/Covariates/Global_2000_2020/PER/ |
| Distance to build settlements (BGSM) | 2017 | 100m | km | ftp://ftp.worldpop.org.uk/GIS/Covariates/Global_2000_2020/PER/ |

Supplementary Table 3 - Summary statistics for covariates included in the analysis

| **Covariate** | **Mean** | **Median** | **Min** | **P1%** | **P99%** | **Max** |
| --- | --- | --- | --- | --- | --- | --- |
| Altitude | 1373.00 | 487.00 | 0.00 | 6.60 | 4350.00 | 4876.00 |
| Distance to health facilities | 0.02 | 0.01 | 0.00 | 0.00 | 0.09 | 0.31 |
| Travel time to health facilities | 14.67 | 2.21 | 0.00 | 0.00 | 219.00 | 888.00 |
| Travel time to cities 50k | 172.50 | 44.49 | 0.51 | 0.51 | 1438.00 | 3269.00 |
| Enhanced vegetation index | 2025.00 | 1771.00 | -3000.00 | -1666.00 | 5581.00 | 6378.00 |
| Urbanicity | 15.65 | 0.36 | 0.00 | 0.00 | 92.99 | 96.95 |
| Nighttime lights | 13.67 | 1.85 | -0.07 | -0.03 | 75.00 | 85.00 |
| Improved water | 0.96 | 0.98 | 0.01 | 0.37 | 1.00 | 1.00 |
| Improved sanitation | 0.63 | 0.75 | 0.00 | 0.00 | 0.98 | 0.99 |
| % of households in Q1 or Q2 | 0.49 | 0.45 | 0.00 | 0.00 | 0.99 | 0.99 |
| Mean number of household members | 3.84 | 3.82 | 2.35 | 2.72 | 4.84 | 5.45 |
| Distance to built settlements | 1.14 | 0.33 | -2.17 | -1.78 | 13.90 | 64.00 |
| Distance to protected areas | 705.00 | 702.00 | 56.00 | 195.00 | 1240.00 | 1278.00 |
| Years of education (women) | 10.00 | 10.40 | 4.10 | 5.30 | 13.60 | 14.00 |
| % of indigenous population | 0.07 | 0.00 | 0.00 | 0.00 | 0.99 | 0.99 |

Note: Zeros and negative values were replaced by a positive value close to 1 for some covariates

Supplementary Table 4 - Covariates selected for each modeled indicator

| **Covariate** | **BCG** | **DPT3** | **MSL** | **CAREP** | **ORS** | **SBA3** | **ANC4** | **FPSmo** |
| --- | --- | --- | --- | --- | --- | --- | --- | --- |
| Altitude |  | X |  |  | X | X |  |  |
| Distance to health facilities |  |  | X |  | X |  | X | X |
| Travel time to health facilities |  |  |  |  | X |  |  |  |
| Travel time to cities 50k |  | X | X |  |  | X |  |  |
| Enhanced vegetation index | X |  | X |  | X | X |  |  |
| Urbanicity |  |  |  |  |  | X |  |  |
| Nighttime lights |  | X |  |  | X | X |  | X |
| Improved water |  |  |  |  |  |  |  |  |
| Improved sanitation | X | X | X |  | X | X | X | X |
| % of households in Q1 or Q2 |  | X |  | X |  |  | X | X |
| Mean number of household members | X |  | X |  |  | X | X |  |
| Distance to built settlements |  |  | X |  |  | X |  |  |
| Distance to protected areas |  |  | X |  | X | X | X |  |
| Years of education (women) | X | X |  |  | X | X | X | X |
| % of indigenous population |  | X |  |  |  |  | X | X |

*BCG* Bacillus Calmette-Guérin vaccine; *DPT3* 3 doses of Diphteria, pertussis, tetanus vaccine; *CAREP* Care-seeking for pneumonia; *SBA* Skilled attendant at delivery; *ANC4* Antenatal care 4 or more visits; *ORS* Oral rehydration salts; *FPSmo* Demand for family planning satisfied by modern methods

Supplementary Table 5 – Model validation metrics

|  | **Cross-validation** | | | **In-sample** | | |
| --- | --- | --- | --- | --- | --- | --- |
| **Indicator** | **Correlation** | **Bias** | **MAE** | **Correlation** | **Bias** | **MAE** |
| BCG | 0.31 | 0.00 | 0.07 | 0.61 | 0.00 | 0.06 |
| DPT3 | 0.20 | 0.00 | 0.18 | 0.55 | 0.00 | 0.16 |
| Measles | 0.20 | 0.00 | 0.22 | 0.59 | 0.00 | 0.20 |
| CAREP | 0.03 | 0.00 | 0.30 | 0.80 | 0.00 | 0.25 |
| SBA | 0.73 | 0.00 | 0.04 | 0.88 | 0.00 | 0.03 |
| ANC4 | 0.25 | 0.00 | 0.06 | 0.45 | 0.00 | 0.06 |
| ORS | 0.26 | 0.00 | 0.27 | 0.60 | 0.00 | 0.24 |
| FPSmo | 0.34 | -0.01 | 0.24 | 0.54 | -0.01 | 0.22 |

*MAE* Mean absolute error; *BCG* Bacillus Calmette-Guérin vaccine; *DPT3* 3 doses of Diphteria, pertussis, tetanus vaccine; *CAREP* Care-seeking for pneumonia; *SBA* Skilled attendant at delivery; *ANC4* Antenatal care 4 or more visits; *ORS* Oral rehydration salts; *FPSmo* Demand for family planning satisfied by modern methods

Supplementary Table 6 – Comparison of predicted vs observed estimates at department-level for the composite coverage index (CCI) and its 8 indicators

| **Department** | **BCG** | | | **DPT3** | | | **MEASLES** | | | **CARE-SEEKING** | | | **ORS** | | | **ANC4** | | | **SBA** | | | **FPSmo** | | | **CCI** | | |
| --- | --- | --- | --- | --- | --- | --- | --- | --- | --- | --- | --- | --- | --- | --- | --- | --- | --- | --- | --- | --- | --- | --- | --- | --- | --- | --- | --- |
|  | **Pred** | **Obs** | **Diff** | **Pred** | **Obs** | **Diff** | **Pred** | **Obs** | **Diff** | **Pred** | **Obs** | **Diff** | **Pred** | **Obs** | **Diff** | **Pred** | **Obs** | **Diff** | **Pred** | **Obs** | **Diff** | **Pred** | **Obs** | **Diff** | **Pred** | **Obs** | **Diff** |
| Amazonas | 94.6 | 93.6 | 1.0 | 84.5 | 83.4 | 1.1 | 85.9 | 81.1 | 4.7 | 66.9 | 65.9 | 1.0 | 27.7 | 24.2 | 3.5 | 94.7 | 92.4 | 2.3 | 85.7 | 81.3 | 4.4 | 66.0 | 61.2 | 4.7 | 72.7 | 69.6 | 3.1 |
| Ancash | 97.6 | 98.0 | -0.4 | 87.6 | 90.0 | -2.4 | 83.3 | 89.0 | -5.7 | 69.6 | 74.5 | -4.8 | 26.6 | 19.1 | 7.5 | 97.7 | 98.2 | -0.5 | 97.9 | 98.0 | -0.2 | 64.7 | 63.0 | 1.8 | 74.9 | 74.9 | 0.0 |
| Apurímac | 97.2 | 99.2 | -2.0 | 89.0 | 90.3 | -1.3 | 82.9 | 82.6 | 0.3 | 68.4 | 75.6 | -7.2 | 23.7 | 22.9 | 0.9 | 96.8 | 97.7 | -0.9 | 99.1 | 99.9 | -0.7 | 59.8 | 61.5 | -1.7 | 73.3 | 75.0 | -1.7 |
| Arequipa | 97.8 | 98.6 | -0.8 | 91.9 | 93.9 | -2.1 | 80.5 | 78.4 | 2.1 | 69.7 | 80.5 | -10.8 | 30.3 | 30.8 | -0.5 | 96.7 | 96.3 | 0.4 | 98.8 | 99.0 | -0.1 | 71.0 | 67.3 | 3.7 | 77.3 | 77.9 | -0.6 |
| Ayacucho | 95.7 | 93.8 | 1.8 | 88.2 | 87.6 | 0.6 | 81.0 | 84.9 | -3.9 | 68.6 | 59.4 | 9.2 | 22.9 | 22.0 | 1.0 | 95.6 | 95.2 | 0.4 | 97.5 | 98.3 | -0.8 | 53.2 | 56.8 | -3.6 | 71.0 | 70.7 | 0.3 |
| Cajamarca | 94.8 | 95.3 | -0.5 | 87.2 | 90.2 | -3.0 | 85.4 | 91.3 | -5.9 | 67.3 | 62.4 | 4.9 | 21.8 | 21.4 | 0.5 | 95.5 | 95.4 | 0.0 | 87.1 | 87.4 | -0.3 | 56.2 | 57.4 | -1.2 | 70.2 | 70.6 | -0.4 |
| Callao | 96.4 | 96.1 | 0.3 | 81.4 | 82.5 | -1.1 | 81.5 | 78.0 | 3.5 | 73.7 | 74.6 | -0.9 | 40.8 | 40.5 | 0.3 | 97.2 | 96.6 | 0.5 | 99.6 | 99.8 | -0.2 | 73.4 | 72.4 | 1.0 | 78.5 | 78.2 | 0.3 |
| Cusco | 96.5 | 96.1 | 0.4 | 88.4 | 85.9 | 2.5 | 83.2 | 74.7 | 8.5 | 66.5 | 74.8 | -8.3 | 28.1 | 33.3 | -5.2 | 97.1 | 97.9 | -0.8 | 97.5 | 98.2 | -0.7 | 55.1 | 58.2 | -3.1 | 72.2 | 74.0 | -1.8 |
| Huancavelica | 95.6 | 97.8 | -2.2 | 86.9 | 88.7 | -1.8 | 84.7 | 89.0 | -4.4 | 66.1 | 71.6 | -5.5 | 18.3 | 19.5 | -1.2 | 95.1 | 95.9 | -0.7 | 92.9 | 93.1 | -0.3 | 42.9 | 46.6 | -3.7 | 66.9 | 69.4 | -2.5 |
| Huánuco | 95.6 | 96.4 | -0.8 | 89.6 | 92.2 | -2.6 | 84.2 | 86.5 | -2.4 | 65.9 | 64.9 | 1.0 | 22.7 | 27.0 | -4.3 | 96.4 | 96.4 | 0.0 | 94.2 | 96.1 | -1.9 | 60.8 | 66.9 | -6.1 | 72.5 | 75.2 | -2.7 |
| Ica | 97.1 | 97.3 | -0.1 | 83.8 | 80.0 | 3.9 | 87.0 | 88.2 | -1.2 | 73.0 | 85.8 | -12.8 | 39.1 | 38.2 | 1.0 | 97.0 | 97.3 | -0.3 | 99.2 | 99.4 | -0.2 | 67.2 | 65.1 | 2.1 | 77.3 | 78.0 | -0.6 |
| Junín | 96.5 | 95.8 | 0.7 | 89.6 | 91.8 | -2.2 | 80.6 | 81.0 | -0.4 | 63.2 | 60.5 | 2.7 | 29.0 | 23.6 | 5.4 | 95.6 | 96.3 | -0.7 | 89.7 | 91.7 | -1.9 | 62.1 | 64.2 | -2.1 | 72.5 | 72.6 | -0.1 |
| La Libertad | 97.1 | 96.7 | 0.4 | 82.6 | 80.6 | 2.0 | 82.9 | 80.7 | 2.2 | 68.0 | 64.3 | 3.7 | 32.1 | 34.9 | -2.8 | 96.8 | 96.3 | 0.4 | 92.1 | 89.8 | 2.3 | 66.5 | 62.6 | 3.9 | 74.3 | 72.5 | 1.8 |
| Lambayeque | 95.9 | 95.4 | 0.4 | 86.1 | 84.5 | 1.7 | 74.5 | 71.5 | 3.0 | 68.9 | 72.6 | -3.6 | 36.6 | 37.4 | -0.8 | 95.1 | 94.0 | 1.0 | 97.1 | 95.2 | 1.8 | 68.6 | 60.9 | 7.7 | 75.8 | 73.6 | 2.2 |
| Lima Province | 95.2 | 94.9 | 0.3 | 83.6 | 82.8 | 0.8 | 80.3 | 79.0 | 1.3 | 73.1 | 75.5 | -2.4 | 38.4 | 37.4 | 1.0 | 97.3 | 97.6 | -0.2 | 99.6 | 99.5 | 0.0 | 72.6 | 70.2 | 2.4 | 78.1 | 77.5 | 0.6 |
| Loreto | 83.8 | 85.6 | -1.8 | 79.3 | 81.7 | -2.5 | 70.4 | 75.8 | -5.4 | 66.2 | 73.1 | -6.9 | 32.5 | 40.0 | -7.5 | 90.2 | 90.1 | 0.1 | 66.1 | 72.3 | -6.2 | 59.2 | 61.3 | -2.1 | 66.2 | 70.1 | -3.8 |
| Madre de Dios | 95.5 | 99.5 | -4.0 | 81.0 | 82.2 | -1.2 | 71.8 | 73.8 | -1.9 | 61.7 | 52.2 | 9.5 | 31.2 | 45.7 | -14.6 | 93.4 | 94.7 | -1.2 | 88.6 | 97.9 | -9.3 | 54.5 | 65.4 | -10.9 | 68.6 | 73.8 | -5.2 |
| Moquegua | 98.0 | 98.3 | -0.3 | 90.1 | 88.8 | 1.4 | 85.9 | 88.8 | -2.9 | 70.1 | 77.0 | -6.9 | 29.4 | 36.3 | -6.8 | 97.4 | 97.9 | -0.5 | 97.7 | 99.5 | -1.8 | 69.2 | 72.2 | -3.0 | 76.9 | 79.7 | -2.8 |
| Pasco | 96.3 | 95.3 | 1.0 | 85.6 | 83.9 | 1.7 | 82.3 | 81.0 | 1.3 | 65.6 | 71.9 | -6.3 | 22.5 | 31.2 | -8.7 | 96.2 | 96.7 | -0.5 | 93.6 | 96.8 | -3.3 | 65.4 | 69.9 | -4.5 | 72.9 | 76.1 | -3.1 |
| Piura | 97.2 | 98.0 | -0.8 | 85.7 | 87.5 | -1.8 | 81.1 | 81.8 | -0.7 | 67.4 | 69.3 | -1.9 | 36.8 | 37.5 | -0.7 | 96.6 | 96.8 | -0.2 | 91.9 | 91.9 | 0.0 | 71.9 | 70.8 | 1.1 | 76.4 | 76.8 | -0.4 |
| Puno | 94.5 | 91.4 | 3.1 | 76.3 | 75.0 | 1.4 | 72.7 | 70.9 | 1.9 | 64.4 | 40.8 | 23.6 | 21.3 | 18.6 | 2.7 | 92.5 | 91.9 | 0.6 | 92.5 | 95.0 | -2.5 | 41.2 | 43.4 | -2.1 | 64.1 | 61.1 | 3.0 |
| San Martín | 94.6 | 93.2 | 1.4 | 90.2 | 90.9 | -0.7 | 82.7 | 78.5 | 4.1 | 64.0 | 50.0 | 14.0 | 39.4 | 42.1 | -2.7 | 97.5 | 98.1 | -0.6 | 93.5 | 92.0 | 1.5 | 66.2 | 66.5 | -0.3 | 75.7 | 74.0 | 1.7 |
| Tacna | 98.5 | 99.3 | -0.8 | 90.2 | 90.2 | 0.0 | 81.7 | 78.9 | 2.9 | 70.9 | 64.8 | 6.0 | 32.1 | 21.2 | 10.9 | 97.5 | 97.7 | -0.2 | 98.2 | 98.6 | -0.4 | 63.6 | 60.0 | 3.6 | 75.8 | 72.7 | 3.1 |
| Tumbes | 98.0 | 98.4 | -0.4 | 92.9 | 93.9 | -1.1 | 87.2 | 89.7 | -2.4 | 63.9 | 67.9 | -4.1 | 43.8 | 51.1 | -7.3 | 96.5 | 96.7 | -0.2 | 98.4 | 99.1 | -0.6 | 85.2 | 83.2 | 2.0 | 82.3 | 83.6 | -1.3 |
| Ucayali | 92.7 | 94.5 | -1.8 | 84.7 | 87.1 | -2.4 | 70.2 | 69.1 | 1.0 | 61.4 | 60.8 | 0.6 | 41.5 | 42.0 | -0.5 | 92.0 | 94.0 | -2.1 | 82.5 | 88.6 | -6.2 | 66.3 | 65.9 | 0.4 | 72.0 | 73.3 | -1.3 |

*BCG* Bacillus Calmette-Guérin vaccine; *DPT3* 3 doses of Diphteria, pertussis, tetanus vaccine; *CAREP* Care-seeking for pneumonia; *SBA* Skilled attendant at delivery; *ANC4* Antenatal care 4 or more visits; *ORS* Oral rehydration salts; *FPSmo* Demand for family planning satisfied by modern methods; *CCI* Composite coverage index

Supplementary table 7 – Predicted estimates for the composite coverage index (CCI) for the provinces of Peru

| **Provinces** | **Point estimate** | **Standard error** | **2.5th percentile** | **97.5th percentile** |
| --- | --- | --- | --- | --- |
| Abancay | 75.9 | 4.3 | 66.7 | 83.7 |
| Acobamba | 66.9 | 2.2 | 62.4 | 71.0 |
| Acomayo | 72.3 | 2.8 | 66.5 | 77.6 |
| Aija | 70.2 | 2.5 | 65.0 | 74.8 |
| Alto Amazonas | 61.9 | 1.7 | 58.6 | 65.1 |
| Ambo | 72.4 | 1.7 | 69.0 | 75.6 |
| Andahuaylas | 71.8 | 1.4 | 69.0 | 74.4 |
| Angaraes | 67.1 | 2.0 | 62.9 | 70.7 |
| Anta | 73.2 | 1.9 | 69.4 | 76.6 |
| Antabamba | 71.7 | 2.0 | 67.8 | 75.6 |
| Antonio Raymondi | 69.9 | 2.2 | 65.5 | 74.2 |
| Arequipa | 77.5 | 2.1 | 73.3 | 81.3 |
| Ascope | 76.8 | 1.9 | 73.0 | 80.3 |
| Asunción | 71.9 | 2.4 | 66.9 | 76.7 |
| Atalaya | 60.2 | 2.5 | 55.0 | 64.9 |
| Ayabaca | 69.8 | 2.0 | 65.9 | 73.6 |
| Aymaraes | 73.1 | 1.7 | 69.8 | 76.2 |
| Azángaro | 61.6 | 2.0 | 57.5 | 65.4 |
| Bagua | 73.6 | 2.0 | 69.5 | 77.2 |
| Barranca | 79.4 | 2.5 | 74.2 | 84.0 |
| Bellavista | 72.8 | 1.9 | 69.0 | 76.3 |
| Bolognesi | 71.5 | 1.8 | 68.1 | 74.9 |
| Bolívar | 63.3 | 2.4 | 58.5 | 68.0 |
| Bongará | 73.5 | 2.7 | 67.8 | 78.5 |
| Cajabamba | 66.9 | 2.0 | 63.1 | 70.8 |
| Cajamarca | 69.8 | 2.0 | 65.8 | 73.4 |
| Cajatambo | 71.4 | 2.2 | 67.1 | 75.4 |
| Calca | 71.6 | 2.0 | 67.5 | 75.3 |
| Callao | 78.5 | 2.8 | 72.1 | 83.8 |
| Camaná | 79.5 | 2.3 | 74.9 | 83.9 |
| Canas | 69.6 | 2.2 | 65.1 | 73.7 |
| Canchis | 70.8 | 4.3 | 61.9 | 78.9 |
| Candarave | 70.8 | 2.4 | 65.8 | 75.6 |
| Cangallo | 67.5 | 1.8 | 63.9 | 70.8 |
| Canta | 74.3 | 3.2 | 67.3 | 79.8 |
| Carabaya | 66.5 | 2.2 | 62.3 | 70.9 |
| Caravelí | 76.4 | 2.3 | 71.6 | 80.6 |
| Carhuaz | 72.4 | 2.3 | 67.7 | 76.6 |
| Carlos Fermin Fitzcarrald | 70.8 | 2.2 | 66.5 | 75.1 |
| Casma | 76.0 | 2.2 | 71.6 | 80.1 |
| Castilla | 76.1 | 2.4 | 71.1 | 80.3 |
| Castrovirreyna | 63.6 | 2.5 | 58.7 | 68.4 |
| Caylloma | 70.6 | 2.0 | 66.5 | 74.3 |
| Cañete | 76.5 | 2.4 | 71.6 | 80.9 |
| Celendín | 67.5 | 2.0 | 63.5 | 71.2 |
| Chachapoyas | 77.3 | 4.6 | 68.2 | 85.3 |
| Chanchamayo | 74.3 | 4.7 | 64.0 | 82.3 |
| Chepén | 77.6 | 4.0 | 69.4 | 84.4 |
| Chiclayo | 77.0 | 2.7 | 71.4 | 81.8 |
| Chincha | 77.3 | 4.0 | 68.9 | 84.2 |
| Chincheros | 71.2 | 2.7 | 65.7 | 76.2 |
| Chota | 69.1 | 1.5 | 65.9 | 72.0 |
| Chucuíto | 59.9 | 2.5 | 55.0 | 64.8 |
| Chumbivilcas | 69.9 | 1.8 | 66.4 | 73.3 |
| Chupaca | 71.5 | 2.9 | 65.8 | 76.9 |
| Churcampa | 66.1 | 1.7 | 62.6 | 69.3 |
| Concepción | 71.0 | 3.0 | 65.0 | 76.5 |
| Condesuyos | 74.3 | 2.3 | 69.7 | 78.6 |
| Condorcanqui | 54.8 | 2.4 | 50.3 | 59.3 |
| Contralmirante Villar | 80.7 | 1.7 | 77.1 | 83.8 |
| Contumazá | 71.1 | 2.1 | 66.8 | 74.9 |
| Coronel Portillo | 74.2 | 2.9 | 68.1 | 79.7 |
| Corongo | 67.0 | 2.6 | 61.8 | 71.6 |
| Cotabambas | 72.7 | 1.7 | 69.5 | 75.9 |
| Cusco | 73.9 | 4.4 | 65.1 | 82.0 |
| Cutervo | 71.2 | 2.3 | 66.4 | 75.8 |
| Daniel Alcides Carrión | 71.9 | 1.6 | 68.7 | 74.9 |
| Dos de Mayo | 69.4 | 1.9 | 65.8 | 73.2 |
| El Collao | 62.9 | 2.8 | 57.0 | 68.3 |
| El Dorado | 73.6 | 2.3 | 68.9 | 77.7 |
| Espinar | 67.2 | 2.7 | 61.5 | 72.3 |
| Ferreñafe | 71.2 | 2.1 | 67.1 | 75.3 |
| General Sánchez Cerro | 72.3 | 2.2 | 67.9 | 76.4 |
| Gran Chimú | 70.3 | 3.6 | 62.8 | 76.6 |
| Grau | 72.7 | 2.3 | 68.1 | 76.9 |
| Huacaybamba | 67.5 | 2.2 | 63.2 | 71.8 |
| Hualgayoc | 69.8 | 4.1 | 61.4 | 77.1 |
| Huallaga | 74.4 | 3.4 | 67.2 | 80.8 |
| Huamalíes | 71.7 | 1.5 | 68.8 | 74.3 |
| Huamanga | 72.0 | 4.1 | 64.1 | 79.6 |
| Huanca Sancos | 68.2 | 2.2 | 64.0 | 72.6 |
| Huancabamba | 64.4 | 2.0 | 60.2 | 68.2 |
| Huancane | 61.8 | 2.2 | 57.3 | 66.1 |
| Huancavelica | 67.1 | 2.0 | 63.0 | 70.9 |
| Huancayo | 73.9 | 3.0 | 68.0 | 79.4 |
| Huanta | 68.4 | 1.4 | 65.6 | 71.0 |
| Huaral | 78.1 | 2.0 | 73.8 | 81.9 |
| Huaraz | 74.8 | 4.3 | 65.7 | 82.2 |
| Huari | 71.0 | 1.6 | 67.8 | 74.2 |
| Huarmey | 75.7 | 2.0 | 71.5 | 79.3 |
| Huarochiri | 74.1 | 1.8 | 70.4 | 77.5 |
| Huaura | 77.5 | 2.0 | 73.5 | 81.2 |
| Huaylas | 70.1 | 3.0 | 64.1 | 75.8 |
| Huaytara | 66.3 | 2.1 | 62.3 | 70.2 |
| Huenuco | 75.0 | 2.2 | 70.2 | 79.0 |
| Ica | 77.3 | 3.5 | 70.0 | 83.7 |
| Ilo | 80.4 | 3.4 | 73.3 | 86.8 |
| Islay | 78.6 | 2.2 | 74.3 | 82.4 |
| Jauja | 72.3 | 1.9 | 68.6 | 76.0 |
| Jaén | 72.5 | 1.6 | 69.1 | 75.5 |
| Jorge Basadre | 77.0 | 2.3 | 72.2 | 81.2 |
| Julcan | 62.8 | 2.8 | 56.9 | 68.2 |
| Junín | 72.9 | 1.8 | 69.1 | 76.3 |
| La Convención | 71.7 | 1.7 | 68.0 | 74.9 |
| La Mar | 70.0 | 1.4 | 67.0 | 72.7 |
| La Unión | 72.3 | 2.6 | 67.3 | 77.1 |
| Lago Titicaca | 63.3 | 2.8 | 57.4 | 68.6 |
| Lamas | 76.1 | 2.3 | 71.0 | 80.4 |
| Lambayeque | 73.8 | 1.5 | 70.5 | 76.8 |
| Lampa | 63.3 | 2.1 | 59.1 | 67.3 |
| Lauricocha | 69.4 | 1.9 | 65.3 | 73.2 |
| Leoncio Prado | 75.8 | 1.7 | 72.4 | 78.9 |
| Lima | 78.1 | 1.3 | 75.6 | 80.6 |
| Loreto | 61.7 | 2.1 | 57.8 | 66.1 |
| Lucanas | 72.3 | 1.7 | 69.1 | 75.7 |
| Luya | 72.8 | 1.5 | 69.8 | 75.6 |
| Manu | 68.0 | 2.4 | 63.6 | 72.6 |
| Marañón | 69.2 | 1.7 | 65.7 | 72.4 |
| Mariscal Cáceres | 75.4 | 4.3 | 65.9 | 82.9 |
| Mariscal Luzuriaga | 71.6 | 2.2 | 67.4 | 75.8 |
| Mariscal Nieto | 76.9 | 2.1 | 72.5 | 80.8 |
| Mariscal Ramón Castilla | 62.9 | 2.6 | 57.7 | 68.1 |
| Maynas | 69.1 | 2.0 | 65.0 | 72.7 |
| Melgar | 65.4 | 2.0 | 61.4 | 69.3 |
| Moho | 63.9 | 2.8 | 58.2 | 69.0 |
| Morropón | 73.4 | 1.9 | 69.7 | 77.0 |
| Moyobamba | 76.3 | 3.2 | 69.8 | 81.9 |
| Nazca | 78.7 | 3.0 | 72.2 | 83.9 |
| Ocros | 73.7 | 2.2 | 69.5 | 77.9 |
| Otuzco | 67.0 | 1.9 | 63.2 | 70.7 |
| Oxapampa | 71.0 | 1.6 | 67.8 | 74.0 |
| Oyon | 72.6 | 3.0 | 66.5 | 77.8 |
| Pacasmayo | 76.8 | 2.3 | 71.8 | 81.0 |
| Pachitea | 70.5 | 1.9 | 66.7 | 74.1 |
| Padre Abad | 72.8 | 1.8 | 69.4 | 76.4 |
| Paita | 77.8 | 2.8 | 71.9 | 82.8 |
| Pallasca | 65.2 | 2.5 | 60.0 | 69.8 |
| Palpa | 76.4 | 4.6 | 66.2 | 84.6 |
| Parinacochas | 71.7 | 2.9 | 65.7 | 77.1 |
| Paruro | 72.4 | 1.8 | 68.8 | 75.9 |
| Pasco | 74.7 | 1.5 | 71.6 | 77.4 |
| Pataz | 68.3 | 2.5 | 63.2 | 73.1 |
| Paucar del Sara Sara | 73.1 | 2.4 | 68.3 | 77.7 |
| Paucartambo | 69.0 | 1.8 | 65.1 | 72.7 |
| Picota | 73.6 | 2.5 | 68.7 | 78.3 |
| Pisco | 76.4 | 2.1 | 72.3 | 80.4 |
| Piura | 78.3 | 2.2 | 73.7 | 82.3 |
| Pomabamba | 70.6 | 2.3 | 66.0 | 75.0 |
| Puerto Inca | 70.2 | 1.9 | 66.5 | 73.8 |
| Puno | 64.2 | 3.9 | 56.2 | 71.5 |
| Purús | 55.2 | 3.5 | 48.3 | 62.0 |
| Quispicanchi | 70.5 | 1.9 | 66.7 | 74.0 |
| Recuay | 72.8 | 2.0 | 68.4 | 76.4 |
| Requena | 65.1 | 2.2 | 60.7 | 69.3 |
| Rioja | 73.7 | 1.9 | 69.8 | 77.1 |
| Rodríguez de Mendoza | 75.9 | 1.9 | 72.0 | 79.6 |
| San Antonio de Putina | 62.9 | 2.5 | 58.4 | 67.7 |
| San Ignacio | 71.2 | 1.7 | 67.8 | 74.6 |
| San Marcos | 69.0 | 3.2 | 62.4 | 74.7 |
| San Martín | 78.7 | 4.4 | 69.5 | 86.2 |
| San Miguel | 71.2 | 1.9 | 67.2 | 74.9 |
| San Pablo | 71.0 | 3.6 | 63.6 | 77.5 |
| San Román | 65.6 | 6.1 | 53.6 | 76.8 |
| Sandia | 67.4 | 2.3 | 62.7 | 71.8 |
| Santa | 78.2 | 3.0 | 71.9 | 83.7 |
| Santa Cruz | 71.7 | 2.1 | 67.6 | 75.6 |
| Santiago de Chuco | 65.3 | 2.3 | 60.8 | 69.8 |
| Satipo | 68.2 | 2.4 | 63.3 | 72.7 |
| Sechura | 75.4 | 2.4 | 70.6 | 79.9 |
| Sihuas | 69.8 | 2.0 | 65.6 | 73.6 |
| Sucre | 70.9 | 1.9 | 66.9 | 74.8 |
| Sullana | 79.5 | 1.8 | 75.8 | 82.8 |
| Sánchez Carrión | 68.6 | 2.1 | 64.3 | 72.5 |
| Tacna | 76.2 | 2.8 | 70.2 | 81.2 |
| Tahuamanu | 69.0 | 2.4 | 64.0 | 73.6 |
| Talara | 78.8 | 4.1 | 69.7 | 86.5 |
| Tambopata | 68.8 | 1.8 | 64.9 | 72.2 |
| Tarata | 70.2 | 3.5 | 62.5 | 76.8 |
| Tarma | 74.8 | 1.9 | 71.1 | 78.2 |
| Tayacaja | 67.6 | 1.2 | 65.2 | 70.1 |
| Tocache | 73.7 | 1.8 | 69.8 | 77.0 |
| Trujillo | 77.2 | 3.7 | 69.4 | 83.6 |
| Tumbes | 82.5 | 2.6 | 77.0 | 87.3 |
| Ucayali | 64.1 | 2.5 | 59.6 | 69.4 |
| Urubamba | 73.3 | 2.2 | 69.0 | 77.4 |
| Utcubamba | 74.7 | 1.5 | 71.6 | 77.7 |
| Victor Fajardo | 69.2 | 1.8 | 65.5 | 72.7 |
| Vilcas Huamán | 68.2 | 1.9 | 64.6 | 71.8 |
| Viru | 71.8 | 2.9 | 66.2 | 77.4 |
| Yarowilca | 69.2 | 2.4 | 64.4 | 73.9 |
| Yauli | 73.9 | 3.7 | 66.1 | 80.7 |
| Yauyos | 67.0 | 2.0 | 62.9 | 70.6 |
| Yungay | 70.9 | 2.1 | 66.6 | 75.0 |
| Yunguyo | 67.9 | 4.2 | 59.2 | 75.5 |
| Zarumilla | 82.2 | 2.9 | 75.7 | 87.2 |


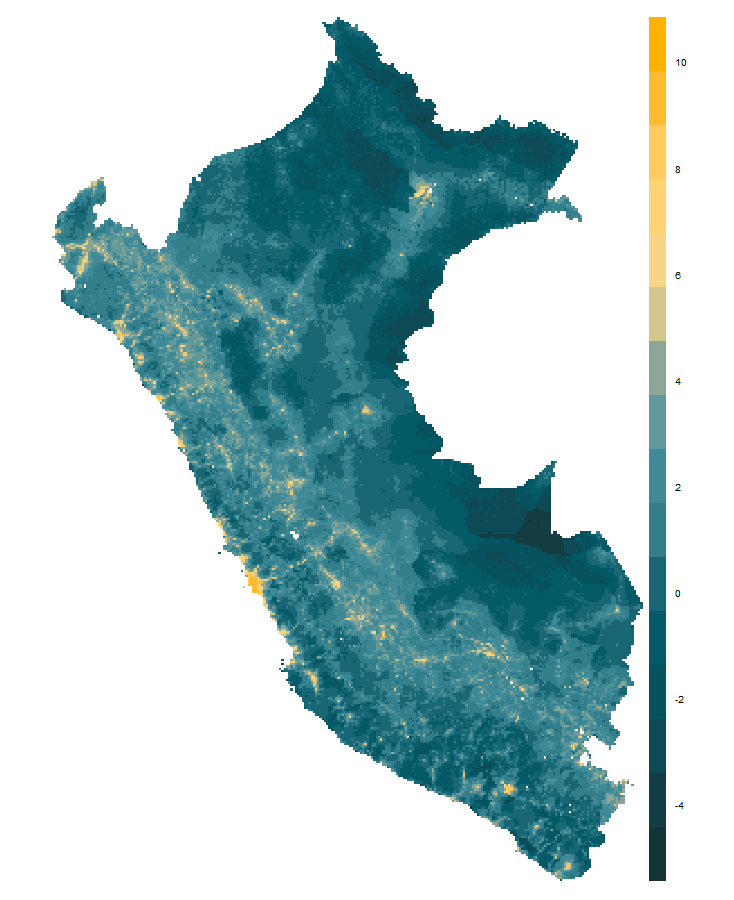


Supplementary Figure 1 – Map of log of population density in Peru


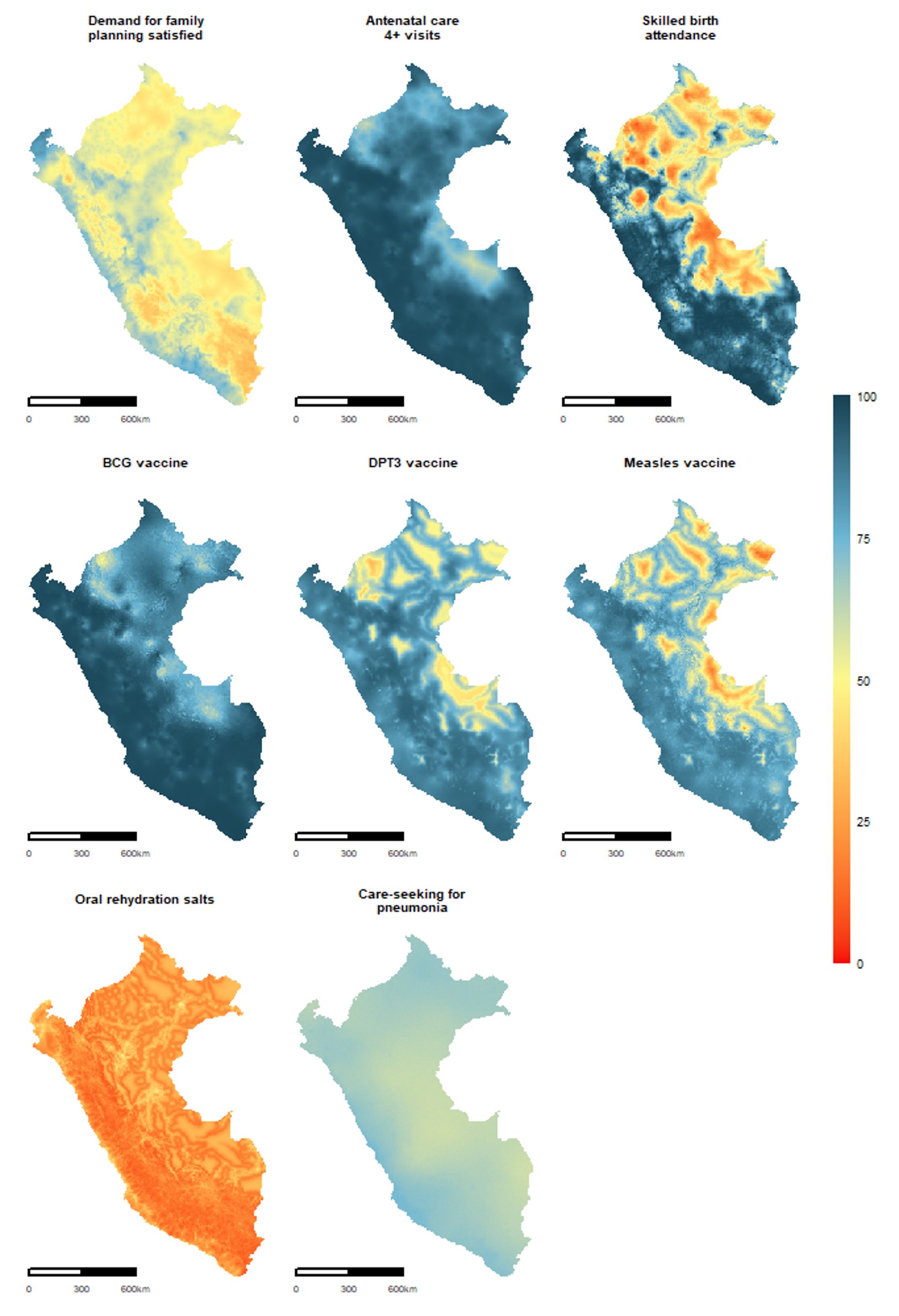


Supplementary Figure 2 – Predicted coverage for all eight composite coverage index (CCI) indicators in Peru
